# Four-year monitoring of the malaria vector *Anopheles funestus* in central-west Cameroon reveals an escalation of pyrethroid resistance combined with high malaria transmission

**DOI:** 10.1186/s12879-026-12708-w

**Published:** 2026-01-28

**Authors:** Hervé Raoul Tazokong, Magellan Tchouakui, Murielle Wondji, Onana Boyomo, Charles Sinclair Wondji

**Affiliations:** 1 Centre for Research in Infectious Diseases (CRID), Medical Entomology Department, P.O. BOX 13591, Yaoundé, Cameroon; 2 Department of Microbiology, Faculty of Science, University of Yaoundé 1, P.O BOX 812, Yaoundé, Cameroon; 3 Vector Biology Department, Liverpool School of Tropical Medicine, Pembroke Place, Liverpool, L35QA UK

**Keywords:** Sporozoite rate, Plasmodium, Anopheles funestus, Insecticide resistance

## Abstract

**Background:**

Insecticide resistance presents a critical obstacle to malaria vector control, necessitating ongoing surveillance to guide control strategy. Despite widespread resistance in central Africa, the temporal adaptive changes driving resistance at both phenotypic and genetic levels remain uncharacterised. This study provides a comprehensive, four-year (2020–2023) assessment of *Anopheles funestus* s.s. in Mibellon, Cameroon, examining sporozoite infection rates and changes in insecticide resistance relative to 2015–2018 data.

**Methods:**

Susceptibility profile, resistance intensity and cone assays were conducted following the WHO protocols. Sporozoite infection was detected in the mosquito head/thorax by TaqMan assay, confirmed by nested-PCR. Gene expression was assessed using RT-qPCR while insecticide resistance markers were genotyped using allele-specific PCR and LNA.

**Results:**

*Plasmodium* sporozoite infection rates ranged from 4 to 21% with the predominance of *P. falciparum* while *P. malariae* and *P. ovale* contributed often as mixed infections. ​Pyrethroid resistance significantly increased over time, with mortalities decreasing from 77.7% in 2015 to 23.2% in 2023 for permethrin and 46.6% in 2016 to just 8.5% in 2023 for deltamethrin, while full susceptibility was noted for organophosphates.​ Worryingly, high intensity of resistance was recorded for all pyrethroids. Partial recovery of susceptibility with PBO suggests other resistance mechanisms beside P450-based metabolic resistance. PBO-based nets yielded high efficacy which decreases slightly over time contrasting with complete loss in efficacy of pyrethroid-only nets. Monitoring the genetic markers revealed a rapid selection of G454A-*CYP9K1* and 4.3 kb SV alleles, which increased considerably and reaching high frequency during the same period in which phenotypic resistance intensified. Other resistance markers (A296S-*rdl* and L119F-*GSTe2*) varied slightly in frequency while the N485I-*Ace1*, 6.5 kb SV, and *CYP6P9a*/*b*_R alleles were absent throughout the years. Consistent overexpression of *CYP9K1* and *CYP6P9a/b* genes in pyrethroid-resistant mosquitoes highlights their potential role in resistance intensification.

**Conclusion:**

The high infection rate and co-circulation of three Plasmodium species coupled with intense pyrethroid resistance pose a serious menace to malaria control in this region. To address these complex challenges, current vector control strategies should prioritize the deployment of PBO-based nets and organophosphates for IRS. Continuous vector and parasite surveillance should guide the choice of future interventions to accelerate progress towards malaria elimination

**Clinical trial:**

Not applicable

## Introduction

Malaria continues to pose significant health challenges to humanity worldwide with an estimated 263 million clinical cases and 597 000 deaths in 2023 [1]. Sub-Saharan Africa is the most affected region, accounting for 95% of global malaria deaths, with children under five and pregnant women being disproportionately affected by the disease [1]. Cameroon is among the 11 high burden countries with 7,343,000 cases and approximately 11,343 deaths accounting for 3.0% and 1.9% of global morbidity and mortality respectively [1]. Effective malaria prevention relies heavily on vector control with the use of insecticides-based interventions comprising long-lasting insecticidal nets (LLINs) and indoor residual spraying (IRS). These two interventions have contributed to significant reduction of malaria between 2000 and 2015 with 663 million of clinical cases averted attributed to LLINs and IRS at 63% and 10%, respectively [2]. However, global progress in malaria control has stalled and even reversed over the last decade. Indeed, malaria cases increased from 231 million in 2015 to 249 million in 2022, while malaria deaths also rose from 586,000 to 608,000 during the same period [1]. This is mainly due to the several biological threats including drug and insecticide resistance [1]. There was confirmed pyrethroid resistance in 55 of 64 malaria endemic countries between 2018 and 2023 [1]. The widespread escalation of insecticide resistance jeopardizes the effectiveness of vector control tools especially LLINs [3, 4] and risks a resurgence in malaria transmission [3, 5].

Insecticide resistance is driven by several mechanisms, including cuticular resistance, behavioral resistance, target-site resistance due to point mutation in the voltage-gated sodium channel (vgsc) and metabolic resistance [6–10]. In *An. gambiae*, the most prevalent and widespread resistance mechanism is the point mutations L1014F/S in the vgsc conferring resistance to DDT and pyrethroids [9, 11, 12]. However, in *An. funestus*, metabolic resistance, driven by the overexpression of detoxification genes like cytochrome P450s, carboxylesterases and glutathione S-transferases is the most prevalent mechanism [10, 13–16]. As recommended by the Global Technical Strategy (GTS), continuous, real-time phenotypic and genetic surveillance is essential to track any change in susceptibility profile and spread of resistance alleles [4] before they cause control failure [3, 4]. So far, monitoring insecticide resistance alleles has been made possible with the detection and design of simple DNA-based resistance markers showing significant geographical variation in the malaria vector *An. funestus*. Indeed, Africa-wide distribution of the L119F-*GSTe2* conferring DDT and pyrethroid resistance in *An. funestus* showed that the allele is fixed in West (Benin) and moderate in Central (Cameroon) and absent elsewhere [17]. Another genetic marker is the N485I-*Ace1*, associated to carbamates resistance and restricted in Southern Africa [18]. Similarly, recent detections of main pyrethroid resistance markers also showed pattern of variation depending of the region in Africa suggesting restriction of gene flow. This includes *CYP6P9a/b*_R [14, 15] and 6.5 kb structural variant (SV) [19] which are predominant in Southern Africa, M220I-*CYP6P4a* and D284E-*CYP6P4b* in West Africa [20], the P450-linked 4.3 kb SV in Central Africa [21] and G454A-*CYP9K1* in East Africa [22]. Similar progress in *An. gambiae* has also led to the design of two metabolic resistance markers: I236M-*CYP6P4* prevalent in East Africa [23, 24] while the E205D-*CYP6P3* is dominant in West/Central Africa [25]. Genetic markers are essential for tracking the spread of resistance alleles in African malaria vectors, assess their impact on bed net efficacy [14, 15, 17, 19, 19–22], and provide timely, evidence-based data to National Malaria Control Programmes (NMCP) for tailored resistance management. Indeed, temporal studies in South, West and East Africa have successfully revealed the rapid selection of key metabolic resistance markers and associated to the loss of efficacy of pyrethroid-only and PBO nets [11, 26–29]. However, most of these studies were conducted at single time point [26, 28, 29] or 3-year monitoring [11, 27] with little attention on the trends of sporozoite infection rate, a key parameter to assess malaria transmission intensity. The long-term dynamics of resistance in Central Africa remain insufficiently characterized. The situation is particularly critical, in central-west Cameroon (Mibellon) because *An. funestus*, the primary malaria vector is perennial, multi-insecticide resistant and subjected to high selective pressure from agricultural pesticides [30–33], making this locality suitable for species-specific vector surveillance. There is critical gap in understanding the temporal evolution and dynamic link between the established resistance markers and shift in susceptibility profile over time. Indeed, sustaining effective malaria vector control depends heavily on robust insecticide resistance monitoring data. This integrated and multi-year temporal study addresses this gap. Therefore, a four-year temporal monitoring in *An. funestus* was conducted in this area between 2020 and 2023 comparing findings to earlier data (2015–2018). The study aims to provide timely, evidence-based data for resistance management by investigating the evolution and dynamic of phenotypic resistance and assessing the changes in efficacy of bed nets over time. Moreover, the trends of resistance allele frequency and gene expression patterns for known genetic markers and metabolic genes were determined, respectively. The *Plasmodium* infection rate was also monitored to track the biological threat in the context of evolving vector resistance. These findings will guide the NMCP to switch immediately from pyrethroid-only to new generation LLINS while implementing active monitoring of the efficacy of the later. Organophosphate could be used for IRS while alternative control measures should be prioritized to curb the emergence of intense pyrethroid resistance in this area.

## Methods

### Study site

**Fig. 1:**
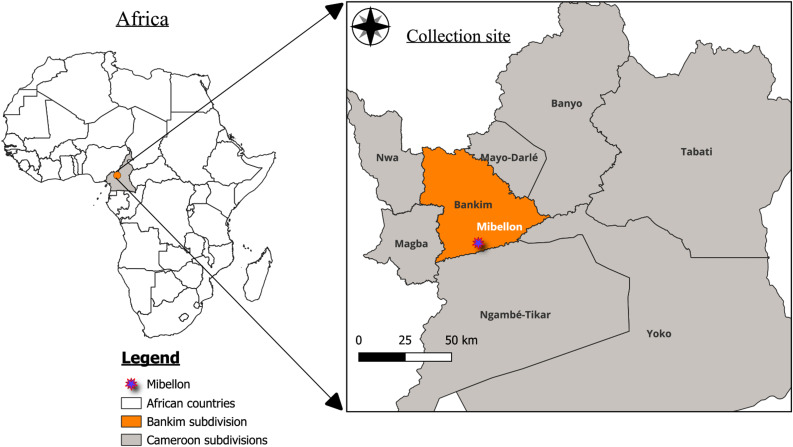
The map of Cameroon showing the study site

A temporal mosquito surveillance was carried out in Mibellon (6°46′N, 11°70′E), central-west Cameroon (Fig. 1) for four years from 2020 to 2023, including December 2020, March 2021, April 2021, May 2021, August 2021, September 2021, April 2022 and October 2023. This locality is a rural village situated in the Adamawa region, Mayo Banyo Division (Bankim sub-division). The Adamawa region is a mountainous area transitioning from Cameroon’s forested south and savanna north. The village is characterised by intense agricultural practices including watermelon, maize and coffee plantations [34]. The use of various insecticides classes in farming has been reported notably pyrethroids, neonicotinoids and carbamates [34]. The climate comprises a bi-modal rainfall pattern characterised by two distinct rainy seasons (August to October and March to April) and two dry seasons (November to February and May to July) [35].

### Mosquito collection and rearing

Prior to collection, verbal consent was obtained from the chief of the village then the head of each household. Earlier in the morning from 5:00 AM to 8:30 AM, indoor resting mosquitoes were collected during five days aided by an electrical aspirator (Prokopack) and kept in a plastic cage. Blood-fed female *Anopheles* mosquitoes were collected gently from the cage using a mouth aspirator then transferred to a paper cup (30 mosquitoes/cup) and supplied with a cotton wool soaked in 10% sugar solution. All the collected mosquitoes were morphologically identified based on the taxonomic keys [36]. The mosquitoes were transported to the laboratory of the Centre for Research in Infectious Diseases (CRID) and kept at the insectary for 3–5 days to allow them to become gravid afterward individual mosquito was introduced in Eppendorf tube to force them to lay eggs [37]. The eggs were transferred in a paper cup containing mineral water till hatching happened. The L1 larvae was randomly mixed in plastic trays for rearing till pupae stage. The larvae were feed with Tetramin^®^ baby fish food, and water was replaced each 2 days. Progeny F_1_ adult mosquitoes were randomly mixed in plastic cage with access to 10% sucrose solution then 3–5 old-days female mosquitoes were used for subsequent bioassays. The insectary was maintained under standard conditions including 25 ± 2 °C and 70 ± 10% relative humidity, and 12 h/12 h light/dark cycle.

### Phenotypic characterisation

#### WHO susceptibility test

Non-blood fed female mosquitoes of 3–5-day old were exposed to each insecticide following the WHO tubes protocol [38]. For each insecticide at least 100 mosquitoes were tested in quadruplicate of 25 individuals per replicate. The insecticides included pyrethroids ([type 1: permethrin 0.75%], [type II: deltamethrin 0.05%, alphacypermethrin 0.05%]), the organochlorine DDT 4%, the carbamates bendiocarb 0.1% and propoxur 0.1% and the organophosphates malathion 5% and pirimiphos-methyl 0.25%. Untreated paper containing silicone oil was included as control in each batch of susceptibility bioassay. Mosquitoes were exposed for 1 h in a WHO tube containing paper impregnated with insecticide and transferred in holding tube supplied with cotton soaked 10% sucrose solution, and mortality were recorded 24 h post-exposure. The susceptible profile of mosquito from 2023 to 2021 was compared to previously published data including 2015, 2016, 2017, 2018 [34, 35].

#### Synergy test

To investigate the involvement of oxidase-based metabolic resistance mechanisms, *Anopheles* mosquitoes were tested using WHO tube bioassays [38] with pre-exposure to piperonyl butoxide (PBO) synergist papers (4%). Mosquitoes were first exposed for 1 h to PBO-impregnated papers, immediately followed by exposure to pyrethroid-impregnated papers [38] for 1 h under standard room conditions (25 ± 2 °C, 70 ± 10% relative humidity). Concomitantly, mosquitoes were exposed to PBO alone and untreated paper as controls. Mortality was recorded 24 h post-exposure. A significant increase in mortality following PBO pre-exposure compared to pyrethroid-only exposure was interpreted as evidence of the involvement of cytochrome P450 monooxygenases in pyrethroid resistance.

#### Intensity of resistance determination

The resistance intensity assay was conducted following the WHO guidelines [38]. Briefly, 20–25 mosquitoes per replicate were exposed to 5x and 10x the diagnostic concentration (DC) of permethrin, deltamethrin and alphacypermethrin. The replicate per dose/insecticide was repeated till obtaining at least 65 alive mosquitoes that were used later to perform gene expression analysis (*n* = 30) and genotype-phenotype association (*n* = 35).

#### WHO cone bioassay

The efficacy of various long-lasting insecticidal nets (LLINs) was assessed following the WHO protocol for cone assay [39]. This includes the pyrethroids-only nets (Olyset, PermaNet 2.0, Interceptor and Duranet), the PBO-based nets (PermaNet 3.0 and Olyset Plus) and the dual active ingredients nets (Royal Guard). Net samples (25 × 25 cm) were taken from the roof and the side panels of each LLIN and mounted on plastic cones. Briefly, 5 non-blood-fed, 2-5-day-old female mosquitoes were introduced in each cone and exposed to the netting for 3 min. A total of 50 mosquitoes were exposed for each net. Mosquitoes were transferred gently to holding paper cups with access to 10% sugar solution, and knock-down was recorded after 1 h, followed by mortality after 24 h. The untreated net was used as negative control. Tests were performed under controlled laboratory conditions (25 ± 2 °C and 70 ± 10% RH).

### Molecular characterisation

#### DNA extraction

Wild-caught (F_0_) mosquitoes were separated into two parts, then DNA was extracted from head/thorax for sporozoite rate determination and abdomen for species identity and genotyping of molecular markers. DNA was also extracted from dead and alive whole individual mosquito from the bioassays for phenotype/genotype correlation. The Livak protocol [40] was used to extract DNA. The extracted DNA was stored in -20 °C and used later to perform molecular analyses.

#### Mosquitoes species identification

Species identification targeting the internal transcribed spacer 2 (ITS2) region of each specific member of the *Anopheles funestus* group was conducted following the protocol of Koekemoer et *al* [41]. , whereas Sine PCR [42] was utilised to identify members of the *An. gambiae* complex.

#### Sporozoite rate determination

The Taqman assay [43] was used to detect the sporozoite stage of *Plasmodium* parasite in the head/thorax samples from each collection period. All the positive samples were subjected to nested PCR developed by Isozumi et al. [44] to confirm and discriminate the four *Plasmodium* species. This technique by targeting the cytochrome c oxidase III (cox3) is highly specific while maintaining high sensitivity. It enables the detection of malaria parasite in the sub-microscopic samples due to the high copy number (20–150) of the mitochondrial gene cox3 per parasite [44]. Therefore, it could outperform all the other nested PCR techniques targeting the 18S rRNA gene (only 4–7 copies/parasite) [45] or the mitochondrial cytochrome b gene [46].

The first step of the PCR comprised: 5 µl BenTaq master mix, 0.34 µl of 10mM Mtu F, 0.34 µl of 10mM Mtu R, 1.25 µl of BSA, 1.57 µl of molecular water giving a total volume of 10 µl and 1.5 µl genomic DNA and amplified as follow: initial denaturation at 94 °C for 30s, 30 cycles of denaturation at 94 °C for 30s, annealing at 63 °C for 1 min, extension at 72 °C for 1 min and final extension at 72 °C for 5 min.

The nested PCR comprised: 5 µl of Bentaq master mix, 0.34 µl of each species-specific primer, 2.82 µl of molecular water and 1.5 µl PCR product from the first step. The final PCR products were separated on a 1.5% agarose gel by electrophoresis and visualized under UV light on a Trans-illuminator machine. The interpretation of the results was as follows: band sizes of 201 bp for *P. falciparum*, 233 bp for *P. malariae*, 204 bp for *P. ovale* and 87 bp for *P. vivax*. Estimation of *Plasmodium* sporozoite infection rate: (total positive samples with nested-PCR/total tested) x 100.

#### Genetic markers genotyping

The frequency of 8 known insecticide resistance markers were surveyed, including: the pyrethroid resistance markers 6.5 kb and 4.3 kb structural variants (SV), *CYP6P9a/b*_R, G454A-*CYP9K1*, the DDT/pyrethroid resistance marker L119F-*GSTe2*, the dieldrin resistance mutation A296S-*rdl* and the N485I-*Ace1* conferring target-site resistance to carbamates.

Allele-specific polymerase chain reaction (PCR) assay was used to genotype the 6.5 kb and 4.3 kb SV [19, 21], L119F-*GSTe2* [17] and G454A-*CYP9K1* [22], while TaqMan allele discriminating technique was used for the detection of A296S-*rdl* [47] and N485I-*Ace1* [18] mutations as previously described. The *CYP6P9a*_R genotyping was done following the restriction fragment length polymorphism (RFLP) PCR protocol [15]. The cis-regulatory *CYP6P9b* variants RFLP PCR technique [14] was modified and amplified using an in house Locked Nucleic Assay (LNA)-based qPCR technique [11].

To assess whether there was any sign of selection occurring at each locus, frequencies of known resistance markers (2011–2017) were collected from previous publications [14, 15, 18, 19, 21, 22, 47] to determine significant increase in allele frequency compared to our first time point of collection in December 2020.

Dead (*n* = 35 individuals) and alive (*n* = 35) mosquitoes upon exposure to pyrethroids were genotyped for the L119F-*GSTe2* mutation, and genotype/phenotype association studies were performed at 1x, 5x and 10x diagnostic concentration for permethrin, deltamethrin and alphacypermethrin insecticides.

#### Gene expression assays

To investigate the potential underlying mechanisms of insecticide resistance over time and in dose response, the expression levels of key detoxifying enzymes were assessed, including *CYP9K1*,* CYP6P9a*,* CYP6P9b*,* CYP6Z1*,* CYP6Z3*,* Carb2514*,* GSTe2* and *CYP6P5* genes. Triplicate of 10 individuals per replicate from survivors to permethrin and alphacypermethrin at 1x, 5x and 10x DCs, as well as unexposed and the lab susceptible Fang was ground in a 1.5 ml Eppendorf tube. Afterward, RNA was extracted using the PicoPure^®^ RNA Isolation Kit (ThermoFisher Scientific, United Kingdom) following the manufacturer’s instructions. cDNA was synthesised using the Superscript III First-Strand Synthesis System (Invitrogen™) following the manufacturer’s instructions. qPCR was processed on the MxPro Machine using gene-specific primers with master mix SYBR Green in a total volume of 10 µL. The expression levels of targeted genes were normalized to those of the housekeeping genes RSP7 and actin, and the relative expression was determined using the 2^−ΔΔCt^ method [48].

### Statistical analyses

The data from October 2023 was compared to the previously published data (from 2015 to 2018) in the same location to assess the pattern and dynamics of insecticide resistance. The susceptibility profile, as well as the intensity of resistance and cone assay results from bioassays, were interpreted following WHO criteria [39, 49]. The chi-square test was used to compare mortality rates between PBO and non-PBO exposure. The visualization was done with GraphPad Prism 8.0 (GraphPad Inc., La Jolla, CA, USA), and the heatmap was plotted using the ggplot2 package in R version 4.4.2. Unpaired Student’s t-test was used to determine the level of significance in gene expression compared to FANG and within phenotypes (unexposed, 1x, 5x and 10x).

To determine whether the sporozoite infection rates differed significantly across sampling months, a logistic regression model was fitted using a generalized linear model (GLM) with a binomial error distribution and a logit link function. The model included the number of positive samples (positive) and negative mosquitoes (total tested - positive) as the response variable, with month as a categorical explanatory variable. This allowed estimation of the odds of mosquito infection across different months while accounting for variation in sample size. The analysis was performed in R v4.4.2 using the glm() function in the stats package.

### Association analysis (Genotype/Survival)

The association of the L119F*-GSTe2* marker and mosquito survival after insecticide exposure was assessed using Fisher’s exact test to determine the significance of differences in genotype proportions between alive and dead mosquito groups. The Odds Ratio (OR), along with 95% Confidence Intervals (CI), was then calculated to quantify the strength of the association between the L119F-*GSTe2* resistance genotype and survival status at 1x, 5x and 10x discriminating concentrations (DCs) for permethrin, deltamethrin and alphacypermethrin.

All tests were considered significant at *p* < 0.05.

## Results

### *Anopheles* species composition over time in Mibellon

**Fig. 2:**
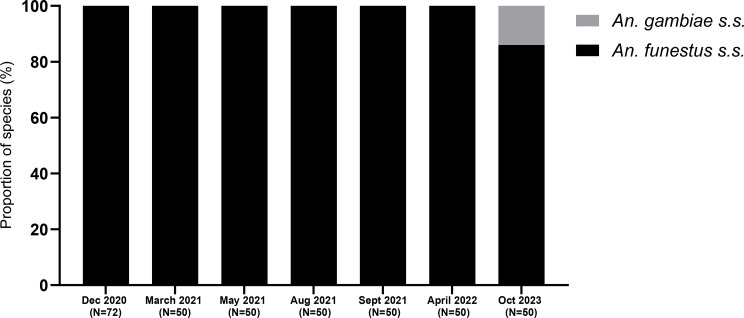
*Anopheles* species composition over time

The species identification revealed that *An. funestus* s.s. was the predominant species over the four years (Fig. 2). Notably, it was found at 100% abundance in all the time points of collection except in October 2023, where 7 *An. gambiae* s.l. were collected and identified molecularly as *An. gambiae* s.s., but due to these very few numbers, they were not included in the rest of the analysis (Fig. 2).

### Temporal dynamics of *Plasmodium* sporozoite infection rates

Overall, a subset of 684 mosquitoes collected over four years was screened for sporozoite infection, with 84 testing positive, resulting in an overall infection rate of 12.3% (Table 1). Among these, *Plasmodium falciparum* was the most prevalent species, detected in 8.2% (56/684) of the total screened samples. *Plasmodium malariae* and *Plasmodium ovale* were less common, with infection rates of 1.46% (10/684) and 1.02% (7/684), respectively. No *Plasmodium vivax* infection was detected. Mixed-species infections were also present among the total screened samples, predominantly *P. falciparum/P. malariae* at 1.31% (9/684). *P. malariae/P. ovale* and *P. falciparum/P. ovale* co-infections each occurred at a prevalence of 0.14% (1/684) (Table 1). Focusing on the 84 positive samples (Table 1), *P. falciparum* accounted for the majority of infections (66.7%, 56/84), while *P. malariae* and *P. ovale* represented 11.9% (10/84) and 8.3% (7/84) of positive infections, respectively. Mixed infections were found in 13.1% (11/84) of positive samples, with *P. falciparum/P. malariae* being the most frequent co-infection (10.7%; 9/84). Other mixed infections (*P. malariae/P. ovale* and *P. falciparum/P. ovale*) constituted 2.4% (2/84) of positive samples (Table 1).

**Fig. 3:**
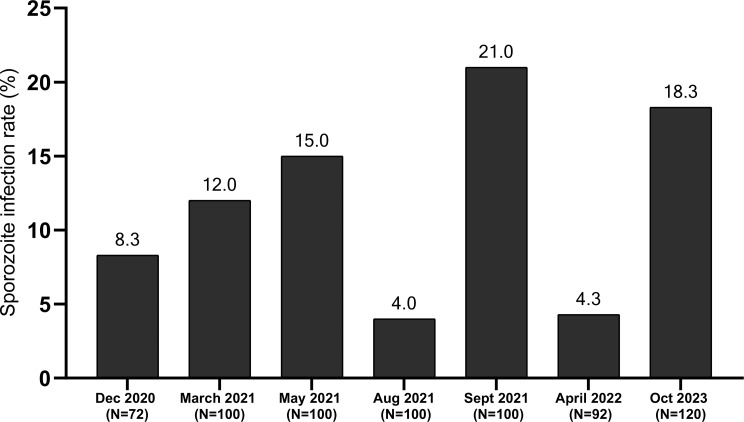
Variation in sporozoite infection rates in *An. funestus* s.s. per period of collection

A binomial logistic regression revealed a significant temporal effect of the sampling month on *Plasmodium* sporozoite positivity (AIC = 42.01). Compared to April 2022, the odds of infection rate were significantly higher in May 2021 (*p* = 0.02), September 2021 (*p* = 0.0019), and October 2023 (*p* = 0.0046), indicating strong temporal variation in sporozoite infection rate. March 2021 showed a marginally higher infection rate (*p* = 0.066) while December 2020 and August 2021 did not differ significantly (*p* > 0.29) (Fig. 3). However, seasonal comparisons in 2021 indicate no significant difference in infection rates between the dry season and the wet season (15% vs. 12.3%, χ² = 0.48, *p* = 0.48). Similarly, when considering all years, the overall sporozoite rate did not differ significantly between dry season and wet season (12.2% vs. 12.3%, χ² = 0.001, *p* = 0.97) (Table 1).

Collectively, these findings underscore the role of the *An. funestus* mosquito as a primary vector sustaining perennial and high malaria transmission in this area, thereby confirming its significant vectorial capacity.

**Table 1:**
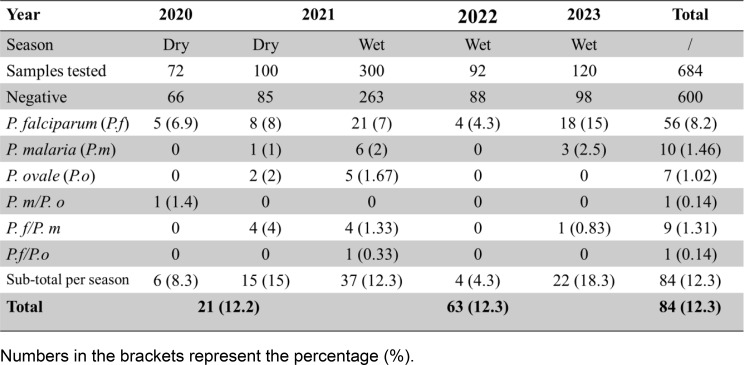
Yearly and seasonal trends in sporozoite infection rates per *Plasmodium* species

### Pattern of insecticide resistance and bed Nets efficacy over time

#### Temporal change in susceptibility profile towards the four main class of insecticides

**Fig. 4:**
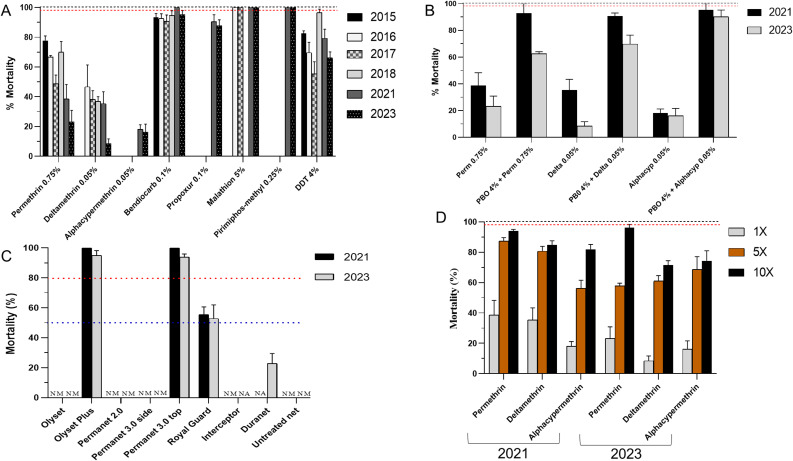
Insecticide resistance monitoring of the malaria vector *An. funestus*. (**A**) Temporal change in susceptibility profile of *An. funestus* between 2015 and 2023 at the discriminating concentration (1x) to the four main public health insecticides. Empty space indicates the insecticide was not tested in this year. (**B**) Impact of the synergist piperonyl butoxide (PBO) on the susceptibility profile, (**C**) Bioefficacy of various long-lasting insecticidal nets, (**D**) Temporal variation of resistance intensity to pyrethroids at 1x, 5x and 10x discriminating concentrations. NM = no mortality, NA = not applicable; the net was not tested. Black dot line indicates 100% mortality, red dot line indicates 98% in tube assays, while blue and red dot lines indicate 50% and 80%, minimal and optimal efficacy, respectively, in cone assay

Insecticide resistance profile of *An. funestus* was monitored between 2021 and 2023. Furthermore, previously published data encompassing four years, including 2015, 2016, 2017 and 2018, were included to assess temporal changes and dynamic of resistance (Fig. 4A).

Regarding pyrethroid insecticides at the diagnostic dose, a significant increase in resistance was observed for permethrin 0.75%, with mortality rates decreasing from 77.7% in 2015 to 23.2% in 2023 (*p* < 0.0001, 95% CI: 41.54–64.53%, χ² = 59.0). Mortalities rates decreased from 66.6% in 2016 to 48.8% in 2017 (*p* = 0.01, 95% CI: 4.1–30.5%, χ² = 6.4) then increased to 69.9% in 2018 before dropping to 38.6% in 2021 (*p* < 0.0001, 95% CI: 17.6–43.0%, χ² = 19.6) (Fig. 4A).

A similar trend of decreasing mortality was observed for deltamethrin, which dropped from 46.6% in 2016 to 8.5% in 2023 (*p* < 0.0001, 95% CI: 26.25–48.62%, χ² = 36.1) indicative of increasing resistance over time. Unlike permethrin, susceptibility to deltamethrin remains relatively stable ranging from 38.4% to 35.55% between 2017 and 2021. However, there was no significant difference in mortality to alphacypermethrin 1x between 2021 (18.07%) and 2023 (16.22%, *p* = 0.7, 95% CI: -8.6-12.3%, χ² = 0.1) although there was no data available back to 2015 as for permethrin and deltamethrin insecticides preventing to capture changes in a timescale of eight years (Fig. 4A).

Mortality rates 24 h post-exposure for the organochlorine DDT at the standard diagnostic dose showed a significant decrease from 79.3% in 2021 to 66.2% in 2023 (*p* = 0.03, 95% CI: 0.7–24.9%, χ² = 4.3). Considering published data revealed a marked decrease in mortality rate from 82.6% in 2015 to 55.3% in 2017 (*p* < 0.0001, 95% CI: 14.58–38.85%, χ² = 17.3) before rising to 96.6% in 2018 (Fig. 4A).

Contrary to DDT, susceptibility to the carbamates bendiocarb and propoxur didn’t vary significantly over time. For example, mortalities ranged from 93.4%, 92.6%, 90.7% to 94.6% in 2015, 2016, 2017 and 2018, respectively, indicating probable resistance towards bendiocarb. However, full susceptibility (100% mortality) was recorded in 2021, then mortality dropped to 95% in 2023 (*p* = 0.02, 95% CI: 0.33–11.1%, χ² = 5.1). Similarly, no significant difference was observed for propoxur with mortalities of 90.5% in 2021 vs. 87.7% in 2023 (*p* = 0.5, 95% CI: -6.1-11.7%, χ² = 0.4). In contrast, susceptibility against the organophosphates remained unchanged between 2016 and 2023 for malathion and between 2021 and 2023 for pirimiphos-methyl, with a 100% mortality rate recorded for both insecticides over the years, indicating full susceptibility (Fig. 4A).

#### Impact of the synergist PBO on mosquito susceptibility profile

In 2023, pre-exposure to PBO significantly increase mortality to permethrin from 23.2% to 62.2% (*p* < 0.0001, 95% CI: 25.5–50.4%, χ² = 30.9) (Fig. 4B). Similar increase in susceptibility were observed for deltamethrin (from 8.46% to 69.81%, *p* < 0.0001) and alphacypermethrin (from 16.22% to 90.12%, *p* < 0.0001) following PBO pre-exposure. While substantial recovery of susceptibility to pyrethroids was observed with the synergist PBO in both years, the level of restoration appeared reduced in 2023 compared to 2021 (Fig. 4B). Importantly, partial restoration was noticed for both years as mortality did not reach 100% suggesting the contribution of others resistance mechanisms beyond cytochrome P450-based metabolism.

#### Efficacy of bed Nets

Evaluation of bed net efficacy revealed persistent loss of efficacy for pyrethroid-only nets. In 2023, no mortality (0%) was recorded for Olyset, PermaNet 2.0, and Interceptor nets upon mosquito exposure, similar to findings from 2021. DuraNet showed minimal efficacy, resulting in only 22% mortality (Fig. 4C). This collectively indicates a drastic loss in the efficacy of these pyrethroid-only nets. The dual-ingredient net Royal Guard (alphacypermethrin and pyriproxyfen) displayed limited efficacy, with average mortalities of 55% in 2021 and 52% in 2023. In contrast, PBO-based nets demonstrated optimal efficacy, yielding high mortalities ranging from 100% to 95.0% for Olyset Plus and from 100% to 93.85% for PermaNet 3.0 top in 2021 and 2023 collections, respectively. This pattern indicates a slight decrease in bed net efficacy in 2023 compared to 2021. Exposure to the PermaNet 3.0 side panel, which contains deltamethrin alone, resulted in 0% mortality, consistent with the low efficacy observed for other pyrethroid-only nets. The untreated net, used as a negative control, resulted in 0% mosquito mortality after 24 h (Fig. 4C).

#### Resistance intensity

Exposure of mosquitoes to higher discriminating doses (5x and 10x) of pyrethroids revealed a high intensity of resistance, as mortality at the 10x dose remained below the 98% threshold for full susceptibility for all tested pyrethroids in both 2021 and 2023. For permethrin at the 5x dose, mortality significantly decrease from 87.3% in 2021 to 57.9% in 2023 (*p* < 0.0001, χ² = 26.0, 95% CI: 18.4–39.6) (Fig. 4D). However, mortality did not vary significantly at the permethrin 10x between 2021 (93.9%) and 2023 (96.1%, *p* = 0.2, χ² = 1.1, 95% CI: -2.2-6.8). For deltamethrin, mortalities significantly decrease between 2021 and 2023 at both the 5x (from 80.7% to 61.0%, *p* < 0.0001, χ² = 18.7, 95% CI: 10.8–28.1) and the 10x DCs (from 84.9% to 71.6%, *p* = 0.001, χ² = 10.4, 95% CI: 5.3–21.2). High resistance intensity was also observed for alphacypermethrin, however, mortalities increase significantly from 56.2% to 68.7% at 5x (*p* = 0.01, χ² = 6.6, 95% CI: 2.9–21.6) whereas a decrease from 82% to 74.3% was observed at 10x (*p* = 0.06, χ² = 3.4, 95% CI: -0.5-15.6) between 2021 and 2023, although this decrease was not statistically significant (Fig. 4D).

### Molecular mechanisms of resistance

#### Dynamic of insecticide resistance markers in field population of *An. funestus*

**Fig. 5:**
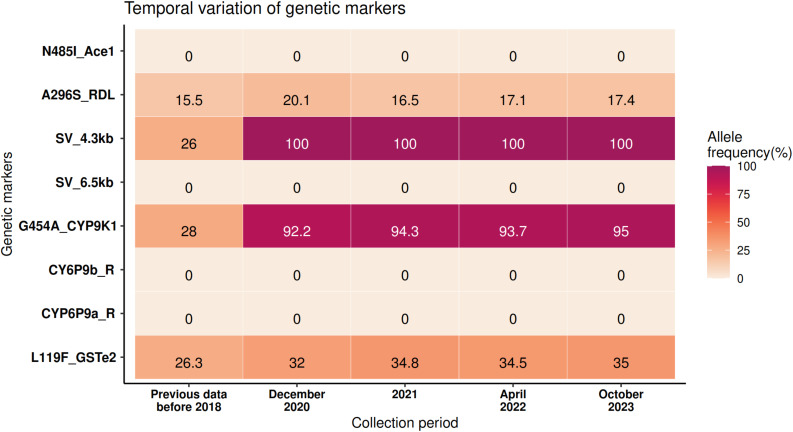
Temporal variation of the frequency of known insecticide resistance markers

The frequency of eight genetic markers previously implicated in conferring resistance to the carbamate bendiocarb (N485I-*Ace1*), the organochlorines (DDT; L119F-*GSTe2* and dieldrin; A296S-*rdl*), and pyrethroids (L119F-*GSTe2*, G454A-*CYP9K1*, *CYP6P9a/b*, the P450-linked structural variants 6.5 kb and 4.3 kb) was monitored. Results indicated the near/complete fixation of G454A-*CYP9K1* allele with frequency ranging from 92.2% in 2020 to 95% in 2023 while the CYP6-linked 4.3 kb SV remained at 100% during the same period. Temporal analysis revealed a marked and rapid selection of these two alleles, whose frequencies increase substantially from 3% to 100% (*p* < 0.0001, χ²=93.2, 95% CI = 85.5–99.3) for 4.3 kb SV + and from 28% to 92.2% (*p* < 0.0001, χ²=42.5, 95% CI = 46.7–75.7) for G454A-*CYP9K1* between 2014 and 2020 respectively (Fig. 5). In contrast, the frequency of the L119F-*GSTe2* allele increased from 26.3% to 32% (*p* = 0.3, χ²=0.8, 95% CI=-6.2-18.6) between 2016 and 2020, remained stable in 2021/2022 and reached only 35% in 2023. A similar pattern was observed with the A296S-*rdl* allele, whose frequency barely increased from 15.5% in 2016 to 20.1% in 2020 (*p* = 0.4, χ²=0.69, 95% CI=-5.7-16.5) and remained relatively stable with 16.5%, 17.1% and 17.4% in 2021, 2022 and 2023, respectively. In opposite, from 2011 and from 2014 to 2020, the resistant alleles of all the others genetic markers, including the 6.5 kb SV, *CYP6P9a_R*,* CYP6P9b_R* and N485I-*Ace1*, were completely absent and remained so till 2023 (Fig. 5).

#### Association of the L119F-*GSTe2* mutation with pyrethroid resistance aggravation

**Fig. 6:**
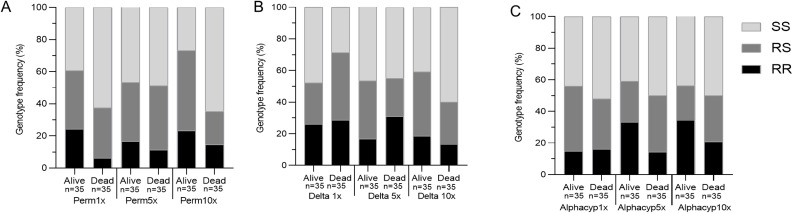
Proportion of different genotypes of the L119F-*GSTe2* mutation in resistance to pyrethroids. Genotype proportions in both alive and dead mosquitoes at 1x, 5x and 10x discriminating concentration to (**A**) Permethrin, (**B**) Deltamethrin and (**C**) Alphacypermethrin. Perm: permethrin, Delta: deltamethrin and Alphacyp: alphacypermethrin

As the L119F-*GSTe2* was the only pyrethroid-linked resistance locus still exhibiting a good segregation of the three genotypes, its association with resistance escalation to pyrethroids was investigated using dead and alive mosquitoes from 2023. In mosquitoes exposed to permethrin 1x, 24.2% of homozygotes resistant (RR), 36.4% of heterozygotes (RS) and 39.4% of homozygous susceptible (SS) were detected in alive. However, in dead mosquitoes, only 6.2% was homozygous RR while the homozygous susceptible SS were more predominant at 62.5%. The survival rate was higher in heterozygous resistant mosquitoes as compared to their homozygous susceptible counterparts (RS versus SS: OR = 2.0, CI = 1.0-3.6, *p* = 0.04, Fisher’s exact test). This was even more pronounced when comparing the RR vs. SS genotypes (OR = 6.4, CI = 2.4–16.5, *p* < 0.0001, Fisher’s exact test) (Fig. 6A; Table 2). Similarly, permethrin 10x survivors were predominantly RR (23%), RS (50%) and less SS (27%) while dead were predominantly SS (65%) and few individuals were RS (20%) and RR (15%) leading to significant odds ratio for RR vs. SS (OR = 3.7; CI = 1.6–7.9; *p* = 0.001, Fisher’s exact test) and RS vs. SS (OR = 6.0; CI = 3.0-11.5; *p* < 0.0001). In contrast, there was no association between the L119F-*GSTe2* mutation and the ability of mosquito to survive to permethrin 5x. There was no statistical significance in all the comparisons of genotypes including RR vs. SS (*p* = 0.28), RR vs. RS (*p* = 0.27) and RS vs. SS (*p* > 0.9). Indeed, there were 17% RR and 46% SS in alive mosquitoes, similar to 11% RR and 49% SS genotypes found in dead mosquitoes exposed to permethrin 5x (Fig. 6A; Table 2). Regarding deltamethrin insecticide, the frequency of RR genotypes was similar in both alive (26%) and dead (28%) at the discriminating concentration 1x. However, there was a heterozygote disadvantage to survive to deltamethrin 1x (RS vs. SS: OR = 0.3; CI = 0.1–0.7; *p* = 0.004, Fisher’s exact test) with 43% RS and 29% SS found in dead mosquitoes whereas 26% RS and 48% SS genotypes were found in alive mosquitoes (Fig. 6B; Table 2). At deltamethrin 5x, there was no significant difference between dead and alive in odds ratios when comparing RR vs. SS and RS vs. SS. However, RR vs. RS comparison indicates negative correlation between deltamethrin 5x dead and alive mosquitoes (OR = 0.3; CI = 0.2–0.8; *p* = 0.01, Fisher’s exact test) (Fig. 6B; Table 2). Similarly, no significant difference in odds ratios was observed when comparing RR vs. SS (*p* = 0.1) in dead and alive at deltamethrin 10x contrasting with the comparison RS vs. SS (OR = 2.2; CI = 1.1–4.2; *p* = 0.01, Fisher’s exact test).

For mosquitoes surviving or dying after exposure to both alphacypermethrin 1x and 10x, none of the comparison between different genotypes yielded a statistical significance suggesting a neutral role of the L119F-*GSTe2* mutation in resistance at these diagnostic concentrations (Fig. 6C; Table 2). Conversely, homozygous resistant RR individuals had 2.8 time more chance to survive after exposure to alphacypermethrin 5x as compared to their susceptible counterparts (OR = 2.8; CI = 1.3–5.8; *p* = 0.006, Fisher’s exact test). Similarly, increased survival to alphacypermethrin 5x was observed in RR vs. RS (OR = 3.2; CI = 1.4–7.3; *p* = 0.004, Fisher’s exact test) but not in RS vs. SS suggesting that survivorship might be associated with carrying the resistant allele at the homozygote stage (Fig. 6C; Table 2).

**Table 2:** Correlation between L119F-*GSTe2* mutation and pyrethroid resistance phenotype. Determined by WHO tube bioassay using F_1_ female *An. funestus* field samples alive and dead to pyrethroid exposure

Samples/Insecticide	Comparison	OR	*p*-value	CI
Permethrin 1x	RR vs. SS	6.4	**< 0.0001**	2.4–16.5
	RR vs. RS	3.4	**0.02**	1.2–9.2
	RS vs. SS	2.0	**0.04**	1.0-3.6
Permethrin 5x	RR vs. SS	1.6	0.28	0.7–3.8
	RR vs. RS	1.7	0.27	0.7-4.0
	RS vs. SS	0.9	> 0.9	0.5–1.8
Permethrin 10x	RR vs. SS	3.7	**0.001**	1.6–7.9
	RR vs. RS	0.6	0.28	0.2–1.4
	RS vs. SS	6.0	**< 0.0001**	3.0-11.5
Deltamethrin 1x	RR vs. SS	0.5	0.1	0.2–1.1
	RR vs. RS	1.5	0.2	0.7-3.0
	RS vs. SS	0.3	**0.004**	0.1–0.7
	RR vs. SS	0.5	0.1	0.2–1.1
Deltamethrin 5x	RR vs. RS	0.3	**0.01**	0.2–0.8
	RS vs. SS	1.4	0.3	0.7–2.7
	RR vs. SS	2.0	0.1	0.8–4.6
Deltamethrin 10x	RR vs. RS	0.9	0.8	0.3–2.1
	RS vs. SS	2.2	**0.01**	1.1–4.2
	RR vs. SS	1.1	0.8	0.5–2.4
Alphacypermethrin 1x	RR vs. RS	0.7	0.5	0.3–1.6
	RS vs. SS	1.5	0.2	0.8–2.7
	RR vs. SS	2.8	**0.006**	1.3–5.8
Alphacypermethrin 5x	RR vs. RS	3.2	**0.004**	1.4–7.3
	RS vs. SS	0.8	0.7	0.4–1.7
Alphacypermethrin 10x	RR vs. SS	1.9	0.06	0.9-4.0
	RR vs. RS	2.3	0.0488	1.1–4.9
	RS vs. SS	0.8	0.7	0.4–1.7

OR: odds ratio, CI: confidence interval, RR: homozygote resistant genotype, RS: heterozygote genotype, and SS: homozygote susceptible genotype. Numbers in bold indicate statistical significance: *p* < 0.05

#### Gene expression: Temporal and dose-response in pyrethroid resistance

**Fig. 7:**
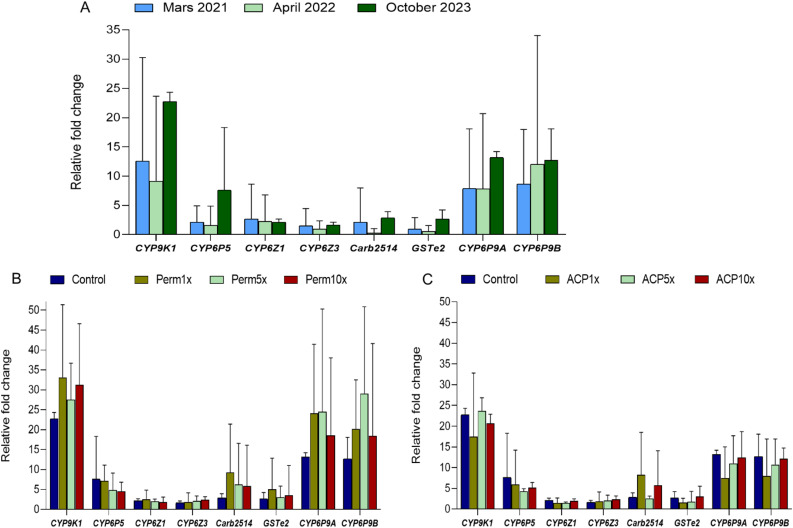
Transcription profile of key detoxifying genes in *An. funestus* population. (**A**) Temporal gene expression in unexposed *An. funestus* between 2021 to 2023. (**B**) Gene expression profile of *An. funestus* in dose response assay including unexposed and survivors to permethrin at 1x, 5x and 10x DCs in October 2023. (**C**) Gene expression profile of *An. funestus* in dose response assay including unexposed and survivors to alphacypermethrin at 1x, 5x and 10x DCs in October 2023. DC: diagnostic concentration, Perm: permethrin and ACP: alphacypermethrin

Temporal analysis (including March 2021, April 2022 and October 2023) of key resistance genes revealed consistently high constitutive overexpression of three cytochrome P450 monooxygenases including *CYP9K1*,* CYP6P9a*, and *CYP6P9b* over a three-year period in unexposed mosquitoes. For instance, *CYP9K1* fold changes (FC) ranged from 12.5 ± 17.7 in March 2021 to 22.1 ± 1.6 in October 2023, displaying no significant temporal variation (one-way ANOVA; df = 8, F = 2.0, *p* = 0.21). The duplicated P450s *CYP6P9a* and *CYP6P9b* genes also exhibited high expression levels, ranging from 7.8 ± 10.2 to 13.1 ± 1.0 and 8.6 ± 9.3 to 12.7 ± 5.3 FC, respectively, throughout the sampling periods. In contrast, other resistance genes tested, including *CYP6P5* (1.6–7.6 FC), *CYP6Z1* (2.1–2.6 FC), *CYP6Z3* (1.0–1.5 FC), *Carb2514* (0.2–2.9 FC), and *GSTe2* (0.5–2.6 FC), exhibited comparatively lower constitutive expression (Fig. 7A).

To further investigate the gene expression profiles under insecticide pressure, a dose-response assay was conducted with increasing concentrations (1x, 5x, and 10x) for both permethrin and alphacypermethrin. This assay demonstrated that all evaluated genes were overexpressed upon exposure, consistent with the temporal data, notably, *CYP9K1*,* CYP6P9a*, and *CYP6P9b* were the most highly upregulated genes (Fig. 7B & C). For *CYP9K1*, expression increased from the unexposed baseline (22.7 ± 1.6 FC) to a peak of 33.1 ± 18.2 FC at permethrin 1x. Although expression slightly decreased at 5 × (27.5 ± 9.1 FC), it rose again at 10 × (31.2 ± 15.3 FC), but there was no clear dose-dependent relationship within the tested range (Fig. 7B & C). Conversely, exposure to alphacypermethrin resulted in a decrease in *CYP9K1* expression from 22.7 FC in unexposed to 17.4 ± 15.3 FC at 1x, and remained relatively stable at higher concentrations (23.6 ± 3.2 FC at 5x and 20.7 ± 2.2 FC at 10x). Similar patterns were observed for *CYP6P9a* and *CYP6P9b*, which showed increased expression at permethrin 1x but decreased expression at alphacypermethrin 1x compared to their unexposed levels (Fig. 7B & C).

## Discussion

Malaria-carrying mosquitoes have an incredible ability to rapidly evolve and adapt to their environment, including developing resistance to insecticides as evidenced in *An. gambiae* [50] and *An. funestus* [51], therefore, it is crucial to monitor any shift in susceptibility profile to adjust and apply smarter resistance management strategies. This study provides critical insights into the dynamics of pyrethroid resistance, the efficacy of bed nets and the persistence of malaria transmission over four years in Mibellon, Cameroon.

### High and persistent sporozoite infection rates: a threat to malaria control

Despite ongoing control interventions such as bed nets extensively deployed in Cameroon [52], a high sporozoite infection rate (SIR: 12.3%) was detected in Mibellon in the primary vector *An. funestus* reaffirming its high vectorial capacity [31, 53]. This species alone has been reported to intensifying malaria transmission, sustaining perennial malaria transmission throughout the year with an entomological inoculation rate of 792 ib/h/year and responsible for 98.6% of all malaria transmission events [31]. The SIR reported here is comparable to previous findings in the same locality, which indicated prevalence ranging from 11% to 13.8% between 2019 and 2021 [31, 32, 54], and similar to the infection rate found in *An. coluzzii* (13%) from Niger [55]. In contrast, the current SIR was more than double of what has been reported in Mozambique [27]. Although, highest infection was found in some months of the wet season, there was no seasonal variation of SIR over the study period [31], aligning with 2.9% and 2.3% found in dry and wet seasons, respectively, in *An. gambiae* s.l. from the forested zone of Ghana [56]. However, back in 2017, only 5% of *An. funestus* was infected by *Plasmodium* sporozoite in Mibellon [33, 34], similar to that of *Anopheles* mosquito population from southern Cameroon [57], indicating a rising risk of malaria parasite transmission over time. Furthermore, in *An. funestus* it has been observed that 119F/F-GSTe2 homozygous resistant mosquitoes were significantly more likely to be infected compared to both heterozygous and homozygous susceptible [33]. However, such approach was impossible in this study because the main pyrethroid resistance markers were fixed (100%) in the field mosquito population from Mibellon preventing any further investigations between these markers and the infection rate. Indeed, reduced *Plasmodium* infection has been reported in homozygous susceptible mosquitoes SV-/SV- to the 4.3 kb insertion as compared to their counterparts homozygous resistant SV+/SV+ [21]. Conversely, Adams and colleagues showed that pyrethroid-resistant *An. gambiae* selected in the lab carried the highest burden and intensity of *Plasmodium falciparum* as compared to their susceptible counterparts, suggesting how phenotype can impact parasite development in the mosquito vector. By contrast, there was no direct association between the intensity of infection and known resistance mechanisms, including *kdr* and overexpression of key cytochrome P450s [58, 59]. Altogether, it is clear that previous studies revealed contradicting findings in regards to association between resistance phenotype/markers and sporozoite infection rate [60–62], warranting further investigations.

The co-occurrence of three *Plasmodium* species, including *P. falciparum*, *P. malariae* and *P. ovale* circulating at appreciable frequencies in natural *An. funestus* population further complicates malaria control in this area [54]. For instance, a decline in *P. falciparum*, the predominant human malaria parasite, might be associated to the rise and spread of non-falciparum species as observed in Eastern Tanzania [63], raising concerns for malaria elimination programmes that traditionally focus on falciparum infections. Altogether, the mixed infections circulating in *An. funestus* mosquito would likely cause co-transmission in a single bite and would further complicate malaria control efforts and delay progress towards elimination. This highlights the need for improved surveillance, diagnostic tools, and tailored intervention strategies addressing these non-falciparum species.

### Pattern of increased levels of pyrethroid resistance over time associated to drastic loss in efficacy of pyrethroids-only nets

Drastic reduced susceptibility to pyrethroids was noticed in temporal analysis. This finding was consistent with intensity bioassays, which revealed elevated levels of resistance to all pyrethroids tested, including permethrin, deltamethrin and alphacypermethrin, with mortality < 95% at the 10x DC, indicating high intensity of resistance. The first report of intense pyrethroid resistance was noted in *An. funestus* s.s. population from Mozambique with > 10% mosquitoes surviving 180 min exposure in time course bioassays [27]. Similar reports in dose-response assays have been documented in *Anopheles gambiae*, *Anopheles funestus* and *An. coluzzii* in several region of Africa including Central Africa (Democratic Republic of Congo and Cameroon) [12, 57, 64, 65], West Africa (Ghana) [29], East Africa (Uganda and Tanzania) [11, 28, 66] and Southern Africa (Malawi) [26, 67]. The current trend of increasing levels of pyrethroid resistance has been also observed in *Culex* mosquitoes with < 20%, < 40% and < 88% mortality rates at 10x to deltamethrin, permethrin and lambda-cyhalothrin, respectively [68, 69]. This trend is also extended to the arboviral disease vector *Aedes aegypti*, which also displayed a high level of pyrethroid resistance in Mexico [70]. Taken together, this indicates a rise and widespread occurrence of intense resistance to pyrethroids encompassing *Anopheles*,* Aedes* and *Culex* mosquitoes, stressing the need for an integrated vector control strategy to defeat vector-borne diseases like malaria but also arboviral diseases. However, human biting behavior of each mosquito species might vary, further limiting the effectiveness of insecticide-based interventions which mainly target indoor resting mosquitoes, while some mosquitoes prefer to bite outdoors as previously reported [31]. Various factors might be at play to exert such high selection pressure on mosquitoes, including bed nets [14, 15], pesticides used in the agricultural settings, environmental factors [71] and extensive domestic insecticides utilization [72], enabling disease vectors to evolve and adapt in time and space. Pesticides from agricultural use may impose additional selection pressure on malaria vectors, suggesting a multisectoral approach between public health stakeholders, agricultural practitioners and private sector.

The observed high level of resistance in this *Anopheles funestus* population severely compromise the efficacy of pyrethroid-only nets. Previous studies reported that pyrethroid-resistant mosquito populations showed greater ability to survive and bloodfeed after exposure to pyrethroid-only nets [30, 73, 74] likely indicating control failure with these nets. Conversely, PBO-pyrethroid nets offer a promising alternative in the study area, as demonstrated by substantial to complete mortality (reaching 100% post-exposure) probably overcoming the P450s-based metabolic resistance predominant is this species [30, 73–76]. This justifies the recent and encouraging shift from pyrethroid-only to PBO-based nets in the Adamawa region (Mibellon) in Cameroon during the last campaign in 2023 [77]. However, the partial restoration of mortality after PBO pre-exposure, combined with reduced PBO net efficacy in 2023 compared to 2021, suggests the emergence of other resistance mechanisms. To maintain effective control, future interventions must prioritize alternative to pyrethroid-only products. Urgent actions will include intensified monitoring of resistance to both PBO nets and new insecticide classes (neonicotinoids and pyrroles) and prioritizing new tools such as dual-active ingredient nets (e.g., Chlorfenapyr-based nets) which have shown greater efficacy against pyrethroid-resistant vectors [73, 74, 78]. Exploring alternative insecticide classes, such as organophosphates via indoor residual spraying (IRS), given that *An. funestus* remained fully susceptible to pirimiphos-methyl and malathion in the study area. Adopting non-insecticidal approaches, such as improved housing to lower transmission risk as demonstrated in Tanzania [79].

### Fixation in allele frequency of existing resistance markers associated to overexpression of detoxification genes are potential drivers of pyrethroid resistance escalation

None of the resistance markers monitored over the four years showed a significant variation in allele frequency. While the 4.3 kb SV and the G454A-*CYP9K1* alleles were fixed, the L119F-*GSTe2*, the A296S-*rdl* were present at moderate frequency, contrasting with the absence of the *CYP6P9a/b_R*, the 6.5 kb SV and the N485I-*Ace1* alleles, supporting previous findings showing that the latter are restricted to mosquitoes from Southern Africa [18]. This suggests restriction of gene flow between mosquitoes from Southern Africa and other African regions [14, 15, 18, 19]. The absence of the target site resistance mechanism conferred by N485I-*Ace*1 [18] mutation contrasts with reduced susceptibility observed toward carbamate and may be indicative of emerging putative detoxification mechanisms, warranting further investigations to aid in the deployment of control measures involving this class of insecticide. Despite dieldrin has been withdrawn from vector control, the mutation A296S-*rdl* persists in the population, although no significant variation was noticed from 2016 to 2023. This may be explained by the fact that the γ-aminobutyric acid (GABA) receptor is a potential secondary target for pyrethroids [80]; also its proximity to the centromere limits recombination [47]. Similar pattern was observed for the L119F-*GSTe2*, although frequency increased slightly over time. This mutation was primary linked to DDT, yet metabolised to a small extern permethrin but not deltamethrin [17] which might agree with the contrasted results obtained with association of this allele in dead and alive after exposure to increased pyrethroid discriminating dose.

Temporal analysis back to 2014 revealed a massive increase in frequency of the pyrethroid resistance markers, namely 4.3 kb SV and the G454A-*CYP9K1* allele, mirroring the rise in phenotypic resistance. This genetic shift is exemplified by the rapid change observed in *An. gambiae* s.l. from Benin, where homozygous resistant (kdr 1014 F/F) individuals increased from 10% in 2011 to 90% in 2024, reflecting critical shifts in pyrethroid susceptibility [50]. Unlike the intense resistance caused by the co-occurrence of multiple mutations at the voltage-gated sodium channel (*vgsc*) in *Aedes* mosquitoes [70, 81], resistance in *An. funestus* is primarily metabolic-based [34, 82, 83]. Gene expression profiling confirmed constitutive, massive overexpression of the duplicated *CYP6P9a/b* and the *CYP9K1* genes. This constitutive upregulation is linked to the fixation of the previously fixed alleles. For instance, the 4.3 kb transposable element has been shown to impact the expression of *CYP6P9a/b* [21] (which were not overexpressed in Cameroon [14, 15] when the SV frequency was low) [21]. Similarly, the expression of *CYP9K1* correlated with fixation of the G454A allele as previously reported [22]. Fixation of the *CYP6P9a/b*_R alleles and the resulting overexpression of both genes has been correlated with a rapid increase in phenotypic resistance in Mozambican [27] and Malawian *An. funestus* populations [26].

This study demonstrates the relevance of using existing genetic markers of resistance for vector monitoring and surveillance. Tracking sporozoite rate in wild caught *An. funestus* provides value as a proxy for epidemiological data. Molecular surveillance using genetic markers offers the promise of rapid and effective surveillance of resistance, allowing malaria control programmes to make informed choices regarding the most effective interventions to deploy. WHO emphasized the intensification of resistance monitoring in malaria vectors to provide real-time data for evidence-based decision making and includes malaria surveillance as a key intervention [1, 4].

## Conclusion

These findings reveal a persistently high rate of *Plasmodium* sporozoite infection, with the co-circulation of *Plasmodium falciparum*, *P. malariae*, and *P. ovale*, including frequent mixed infections. The results underscore the critical role of *Anopheles funestus* as the dominant vector perpetuating perennial malaria transmission in the study area. Given the widespread and intense pyrethroid resistance and the substantial reduction in efficacy of pyrethroid-only nets, there is an urgent need to prioritize alternative interventions, specifically PBO-based nets and organophosphate IRS for effective vector control. Continuous molecular surveillance of both malaria parasites and resistance markers in mosquito populations is essential for accurate detection of transmission patterns and timely adaptation of control strategies. Such evidence-based approaches are vital to strengthening malaria control efforts and advancing towards the goal of elimination.

## Data Availability

All data generated or analysed during this study are included in this manuscript.

## Abbreviations

AIC: Akaike information criterion
CI: Confidence interval
DC: Diagnostic concentration
DNA: Deoxyribonucleic acid
FC: Fold change
GST: Glutathione S-Transferase
GTS: Global technical strategy
IRS: Indoor residual spraying
ITS2: internal transcribed spacer 2
kdr: Knockdown resistance
LLINs: Long lasting insecticidal nets
LNA: Locked Nucleic Assay
NMCP: National malaria control programme
OR: Odds ratio
PBO: Piperonyl butoxide
PCR: Polymerase chain reaction
RNA: Ribonucleic acid
s.l.: sensu lato
s.s.: sensu stricto
SV: Structural variant
vgsc: Voltage-gated sodium channel
WHO: World Health Organization
